# *miR-6076* rs1463411 polymorphisms are associated with bleeding during clopidogrel treatment in patients with acute coronary syndrome

**DOI:** 10.1186/s40001-023-01068-9

**Published:** 2023-02-24

**Authors:** Zhen-Zhen Mo, Zhen Yuan, Yuan-Yuan Peng, Wan-Lu Zhou, Wei Dai, Guo Wang, Jie Tang, Wei Zhang, Bi-Lian Chen

**Affiliations:** 1 Department of Geriatrics, National Geriatrics Clinic Center, Xiangya Hospital, Central South University, 87 Xiangya Road, Changsha, 410008 People’s Republic of China; 2 Department of Clinical Pharmacology, Xiangya Hospital, Central South University, Xiangya Road 87, Changsha, 410008 People’s Republic of China

**Keywords:** Clopidogrel, Bleeding, *miRNA-6076*, *CYP2C19*, Gene polymorphism, Acute coronary syndrome

## Abstract

Bleeding is a major adverse event during clopidogrel treatment in patients with acute coronary syndrome (ACS). However, the potential mechanism affecting bleeding among individuals is unclear. Herein, we investigated the involvement of CYP2C19*2 and CYP2C19*3, as well as 10 miRNA polymorphisms, in bleeding in Chinese patients with ACS during the first year of clopidogrel treatment. The *miR-6076* rs1463411 G polymorphism was significantly associated with the risk of bleeding (*P* < 0.001), and the rs1463411 GT + GG genotype significantly increased the risk of bleeding (adjusted odds ratio, 6.09; 95% confidence interval, 1.09–34.0; *P* < 0.001). Dual luciferase assay showed that *miR-6076* significantly decreased the mRNA expression of *P2RY12* (*P* < 0.05). P2RY12 mRNA and protein levels were significantly lower in cells transfected with *miR-6076-G* than in cells transfected with *miR-6076-T* (*P* < 0.05). The findings indicate that *miR-6076* targets *P2RY12* mRNA and that *miR-6076* rs1463411 T/G polymorphisms differentially regulate P2RY12 mRNA and protein levels in cells. rs1463411 G polymorphism may increase the risk of bleeding during clopidogrel treatment in patients with ACS.

**Supplementary Information:**

The online version contains supplementary material available at 10.1186/s40001-023-01068-9.

## Introduction

Acute coronary syndrome (ACS) is the main cause of disability and death in patients with coronary heart disease. Platelet activation and aggregation are critical in the etiopathogenesis of ACS; thus, antiplatelet therapy is an important treatment strategy [1]. The combination of clopidogrel and aspirin has shown to reduce the risk of recurrent thrombotic events, but it is also associated with an increased risk of bleeding [2, 3].

Clopidogrel is absorbed through the intestinal ABCB1 transporter and metabolized to an active form mainly by CYP2C19 and CYP3A4 in the liver; the active form binds to the P2RY12 receptor mainly found on the platelet membrane and reduces platelet activation and aggregation [4]. Studies have shown that the use of dual antiplatelets, such as clopidogrel combined with aspirin, can considerably reduce ischemic cardiovascular events in patients with ACS [4–6]. However, dual antiplatelet treatment is always accompanied with an increased risk of bleeding. However, the potential mechanism that affects bleeding in Chinese patients with ACS during clopidogrel treatment remains unclear.

A large-scale clinical study showed that the rates of bleeding and fatal bleeding were 5.9–12.5% and 1.3%, respectively, and that the rate of any transfusion with overt bleeding was 1.4–1.5% when patients received clopidogrel for more than 1 year [7, 8]. Previous clinical studies on genetic polymorphisms of *ABCB1, CYP2C19*, and *P2RY12* found that CYP2C19*17 genetic mutations considerably increased the risk of bleeding in Caucasians [9–11]. However, the allele frequency of CYP2C19*17 reported in Chinese studies is approximately 0.64–0.80%, which is considerably lower than that in Caucasians [12, 13]. In addition, CYP2C19*17, a rare gene polymorphism, has not been found to be correlated with bleeding in patients receiving percutaneous coronary intervention treatment owing to its low prevalence in Asians [14]. Meanwhile, the prevalence of CYP2C19*2 and CYP2C19*3 is as high as 36.6% in Chinese patients with coronary heart disease, and studies on these polymorphisms associated with bleeding events among patients with ACS in China are limited [15].

MicroRNAs (miRNAs) are endogenous, single-stranded small noncoding RNAs that have emerged as important regulators of target gene expression by degrading mRNAs or inhibiting mRNA translation [16, 17]. It has been found that some miRNAs may facilitate the diagnosis of ACS [18] and that polymorphisms of miRNAs may affect the function of the target genes [19, 20].

In this study, we aimed to explore the potential mechanism, including gene polymorphisms (some *miRNAs* and CYP2C19*2 and CYP2C19*3) and clinical characteristics, that may affect the risk of bleeding during clopidogrel treatment in Chinese patients with ACS. First, in silico analysis was performed to determine miRNAs that mainly target *ABCB1*, *CYP2C19*, *CYP3A4*, and *P2RY12*, and the genetic polymorphisms of these *miRNAs* and *CYP2C19* were identified among patients with ACS in China. Second, the correlation between these gene variants and bleeding events during clopidogrel treatment was verified among populations with ACS. Finally, a series of cell experiments was carried out to identify the potential mechanism.

## Materials and methods

### Population with ACS

The study was carried out in two hospitals. One cardiologist group was responsible for the recruitment and follow-up of patients in each hospital. The choice of miRNA was based on TargetScan, and the genetic polymorphisms of *miRNAs* were based on data from the National Center for Biotechnology Information. The minor allele frequencies of these genetic polymorphisms were > 0.05 and primarily reported in the Southern Han Chinese population (CHS) and the Han Chinese population in Beijing (CHB).

The enrollment of patients started in 2011 and continued for 5 years. The follow-up of patients was completed in July 2016. In total, 317 patients with ACS were enrolled from the Shijingshan Institute of Hypertension in Beijing and 246 patients from the Xiangya Hospital, Central South University, Changsha city. ACS was diagnosed according to the 2007 ACC/AHA guidelines [21]. The exclusion criteria have been described in our previous study [20]. We defined the bleeding events based on the criteria of the Bleeding Academic Research Consortium (BARC), mainly consisting of type 2, 3, or 5 [22]. This research was approved by the Ethics Committee of Xiangya Hospital, Central South University (Approval number: 201703768) and was conducted in accordance with the tenets of the Declaration of Helsinki. Written informed consent was obtained from the patients or immediate family members.

### Genotyping

Genomic DNA was extracted from whole-blood specimens using a commercial kit according to the manufacturer’s instructions (Omega BioTek, Norcross, GA, USA). Genotyping was performed using Sequenom MassARRAY. Genotyping for CYP2C19*2 (rs4244285), CYP2C19*3 (rs4986893), and genetic polymorphisms of 10 *miRNA*s, namely, *miR-6076* rs1463411, *miR-7515* rs10192411, *miR-2053* rs10505168, *miR-3612* rs1683709, *miR-499b* rs2070960, *miR-4482* rs45596840, *miR-4268* rs4674470, *miR-7157* rs56148568, *miR-5186* rs9842591, and *miR-605* rs2043556, was performed in all patients. The genetic polymorphisms identified in the samples are listed in Additional file 1.

### Dual luciferase reporter gene assay

The targets of *miR-6076* were mRNAs of *CYP2C19* and *P2RY12*, predicted using TargetScan. Luciferase reporter gene analysis was carried out to verify the predictions. The 3′-untranslated region (3′-UTR) of *CYP2C19* and *P2RY12* mRNAs was cloned into the reporter vector pmiR-RB-Report WT or pmiR-RB-Report MUT (RiboBio, Guangzhou, China). The WT-3′-UTR or MUT-3′-UTR vector was then co-transfected with the *miR-6076* mimic or a negative control (NC). Firefly and *Renilla* luciferase activities were analyzed using a Dual Luciferase Reporter Assay System (Promega, Madison, WI, USA) 24 h post transfection following the manufacturer’s instructions.

### Cell culture

Human BEAS-2B cells were obtained from American Type Culture Collection (RRID:CVCL_0168, Manassas, VA, USA). Three recombinant lentivirus plasmids, namely, pre-*miR-6076-T*, pre-*miR-6076-G*, and empty vector with green fluorescence protein, were designed and synthesized by GeneChem (Shanghai, China). The cells were cultured as previously described [20]. When BEAS-2B cells reached 60% confluency, the three recombinant lentivirus plasmids containing pre-*miR-6076-T*, pre-*miR-6076-G*, or empty vector were used for cell transfection following the manufacturer’s instructions. When BEAS-2B cells reached 80–90% confluency, they were subcultured at a ratio of 1:3 and treated with a complete culture medium containing 1 µg/mL purinomycin, after which the green fluorescence was observed under a fluorescence microscope.

### RNA extraction and quantitative reverse transcription–polymerase chain reaction (RT–PCR)

Total RNA was isolated from cultured cells using TRIzol reagent (Thermo Fisher Scientific, Waltham, MA, USA) and reverse transcribed to cDNA using the All-in-one™ First-Strand cDNA Synthesis Kit (GeneCopoeia, Guangzhou, China). The relative expression of miRNA and mRNA was calculated using the 2^−△△Ct^ method. The expression of mRNAs and miRNAs analyzed using qRT-PCR was normalized to that of *GAPDH* and *U6*, respectively. The qRT-PCR primer sequences for *miR-6076* and *U6* were designed and synthesized by RiboBio. The primer sequences for *P2RY12* and *GAPDH* used in this study were as follows: *P2RY12* forward primer, 5′-GTCATCTGGGCATTCATGTTCT-3′, and *P2RY12* reverse primer: 5′-ACCTACACCCCTCGTTCTTAC-3′; human-*GAPDH*-F: 5′-ACAGCCTCAAGATCATCAGC-3′, and human-*GAPDH*-R: 5′-GGTCATGAGTCCTTCCACGAT-3′.

### Western blotting

The cells were washed thrice with phosphate-buffered saline, and the total protein was extracted using radioimmunoprecipitation assay lysis buffer (Beyotime, Shanghai, China). Protein concentrations were determined using the bicinchoninic acid protein assay (Beyotime). The proteins were separated via sodium dodecyl sulfate polyacrylamide gel electrophoresis and transferred onto a polyvinylidene difluoride membrane (Millipore, Boston, MA, USA). The membranes were incubated with the

P2RY12 antibody (1:1000 dilution, RRID:AB_2840841 Affinity Biosciences, Nanjing city, Jiangsu province, China) at 4 °C overnight. After washing thrice with Tris-buffered saline with Tween 20, the membranes were incubated with horseradish peroxidase-conjugated secondary antibodies and visualized using the Enhanced Chemiluminescence Plus detection system (GeneView, Sacramento, CA, USA).

### Statistical analysis

Statistical significance was analyzed using Chi-squared test, Student’s* t* test, and multivariable logistic regression analysis. Continuous data are presented as mean ± standard deviation (SD), and measurement data are expressed as number (percentage). Statistical analyses were performed using the Statistical Package for Social Sciences (version 22.0; SPSS, Inc., Somers, NY, USA) and GraphPad Prism 7.0 software (GraphPad Software, Inc., CA, USA). Statistical significance was set at *P* < 0.05.

## Results

### Clinical characteristics of and bleeding events in patients

A total of 563 individuals (mean age 62.98 ± 11.49 years) were included in this study. Bleeding was observed in 37 patients (6.6%): 21 (56.8%) had BARC type 2 and 16 (43.2%) had BARC type 3. Fatal bleeding complications were not observed among the patients. One patient had two bleeding episodes (gingival hemorrhage and subcutaneous hemorrhage). The majority of bleeding events included epistaxis (15.4%), gingival hemorrhage (30.8%), subcutaneous hemorrhage (25.6%), and gastrointestinal hemorrhage (28.2%). The baseline characteristics of the patients who suffered bleeding or not are shown in Table 1. Patients with ACS who received angiotensin-converting enzyme inhibitor (ACEI)/angiotensin II receptor blocker (ARB) drugs or who were older than 63.24 years had a decreased risk of bleeding (*P* < 0.05).

**Table 1:** Clinical characteristics and bleeding outcomes in Chinese patients with ACS

Characteristics	Total cohort	Bleeding	Non-bleeding	*P*
	*n* = 563	*n* = 37	*n* = 526	
Age (Years)	62.98 ± 11.49	59.38 ± 11.95	63.24 ± 11.42	0.048
Sex (Male)—*No. (%)*	413 (73.4)	32 (86.5)	381 (72.4)	0.082
Risk factors—*No. (%)*				
Familial history of CAD	107 (19.0)	10 (27.0)	97 (18.4)	0.197
Active cigarette smoking	279 (49.6)	20(54.1)	259 (49.2)	0.613
Arterial hypertension	401 (71.2)	22 (59.5)	379 (72.1)	0.131
Hyperlipidemia	302 (53.6)	17 (45.9)	285 (54.2)	0.395
Diabetes mellitus	183 (32.5)	13 (35.1)	170 (32.3)	0.856
Drug therapy—*No. (%)*				
Statin	537 (95.4)	36 (97.3)	501 (95.2)	1
β-Receptor blocker	446 (82.8)	27 (73.0)	439 (83.5)	0.115
ACEI/ARB	354 (62.9)	11 (29.7)	343 (65.2)	< 0.001
CCB	250 (44.4)	13 (35.1)	237 (45.1)	0.350
PPI	68 (12.1)	8 (21.6)	60 (11.4)	0.071
Aspirin	547 (97.2)	36 (97.3)	511 (97.1)	1

*ACS* acute coronary syndrome; *ACEI* angiotensin-converting enzyme inhibitor; *ARB* angiotensin-receptor blocker; *CAD* Coronary artery disease; *CCB* calcium channel blocker; *PPI* proton-pump inhibitor

### Distribution of genotyping and bleeding

The *miR-6076* rs1463411 genotypes observed were as follows: wild-type (TT) in 385 patients (68.4%), heterozygous (GT) in 167 (29.6%), and homozygous (GG) in 11 (2.0%). The allele distribution was as follows: 83.2% T and 16.8% G. No obvious deviation from the Hardy–Weinberg equilibrium was observed in genotype distribution (*P* = 0.142).

**Fig. 1:**
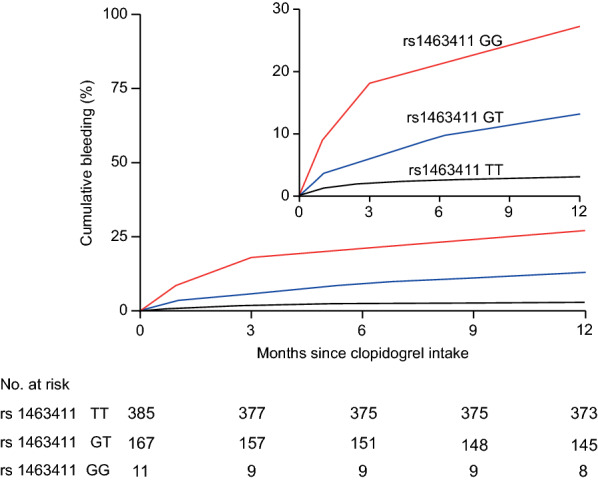
Occurrence of BRAC bleeding among three *miR-6076* rs1463411 genotypes (TT, GT, and GG) during clopidogrel treatment for 12 months

As shown in Table 2, the number of ACS patients with rs1463411 genotypes with bleeding events was 12 (3.1%) for the TT genotype, 22 (13.2%) for the GT genotype, and 3 (27.3%) for the GG genotype. No significant associations were observed between polymorphisms of CYP2C19*2, CYP2C19*3, or the other 9 *miRNA* variant genes and bleeding events (*P* > 0.05). Compared with rs1463411 TT, the rs1463411 GT and GG genotypes significantly increased the risk of bleeding (*P* < 0.001), with the rs1463411 GG genotype resulting in a significantly higher risk of bleeding than the rs1463411 GT genotype (*P* < 0.001; Fig. 1). The rs1463411 GG + GT genotype significantly increased the risk of bleeding (odds ratio [OR] = 5.08, 95% confidence interval [95% CI] 2.49–10.37, *P* < 0.001), especially after adjusting for clinical characteristics (adjusted OR = 5.13, 95% CI 2.48–10.62, *P* < 0.001) among patients with ACS receiving clopidogrel treatment. Using ACEI/ARB drugs significantly reduced the risk of bleeding (OR = 0.23, 95% CI 0.1–0.47, *P* < 0.001), even after adjusting for rs1463411 polymorphisms (OR = 0.24, 95% CI 0.11–0.50,* P* < 0.001).

**Table 2:** Distribution of different *miR-6076* and *CYP2C19* genotypes and clinical characteristics among patients experiencing bleeding during clopidogrel treatment

Variable		Bleeding	Non-bleeding	*P*
		*No. (%)*	*No. (%)*	
CYP2C19*2	GG	18 (6.5)	257 (93.5)	0.901
rs4244285	GA	16 (6.8)	218 (93.2)	
	AA	2 (4.3)	45 (95.7)	
CYP2C19*3	GG	33 (6.3)	489 (93.7)	0.368
rs4986893	GA	4 (10.0)	36 (90.0)	
	AA	0	1 (100.0)	
*miR-6076*	TT	12 (3.1)	373 (96.9)	< 0.001
rs1463411	GT	22 (13.2)	145 (86.8)	
	GG	3 (27.3)	8 (72.7)	
ACEI/ARB		11 (3.1)	343 (96.9)	< 0.001

*miRNA-6076* rs1463411 G resulted in an increased risk of bleeding, in addition taking ACEI/ARB drugs reduced the risk of bleeding significantly

Of the 37 patients with bleeding events, 21 had BARC type 2 and 16 had BARC type 3 events. Among the 385 patients with the rs1463411 TT genotype, 10 had BARC type 2 bleeding and 2 had BARC type 3 bleeding. Among the 178 patients with the rs1463411 GT + GG genotype, 11 had BARC type 2 bleeding and 14 had BARC type 3 bleeding. The rs1463411 GT + GG genotype increased the risk of BARC type 3 bleeding (OR = 6.36, 95% CI 1.15–35.23, *P* = 0.034), which remained significant even after adjusting for clinical characteristics (adjusted OR = 6.09, 95% CI 1.09–34.00, *P* = 0.04).

### miR-6076 targets the 3ʹ-UTR of P2RY12 mRNA

**Fig. 2:**
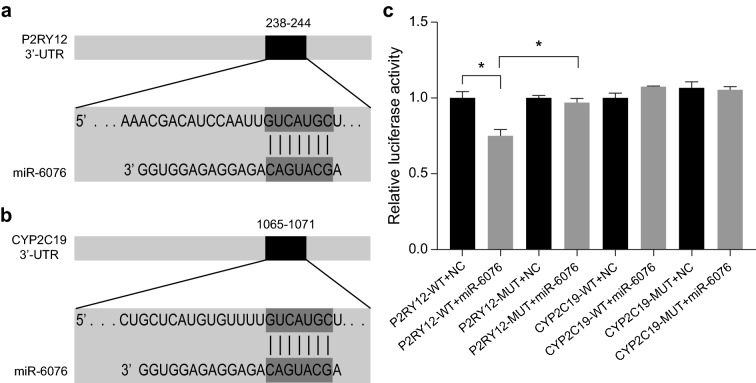
Dual luciferase assay showing *miR-6076* decreasing the mRNA expression of *P2RY12*

As shown in Fig. 2a, b, *miR-6076* was predicted to target the 3ʹ-UTR of the mRNA of *P2RY12* and *CYP2C19* using TargetScan. Luciferase reporter plasmids were cloned with wild-type *P2RY12* and *CYP2C19* 3′-UTRs (WT-3′-UTRs), and a reporter analysis was carried out in HEK293 cells. The dual luciferase analysis showed that the activity of the *P2RY12* 3ʹ-UTR reporter gene was significantly decreased by *miR-6076* (Fig. 2c; *P* < 0.05).

### rs1463411 T/G polymorphisms modulate P2RY12 expression

**Fig. 3:**
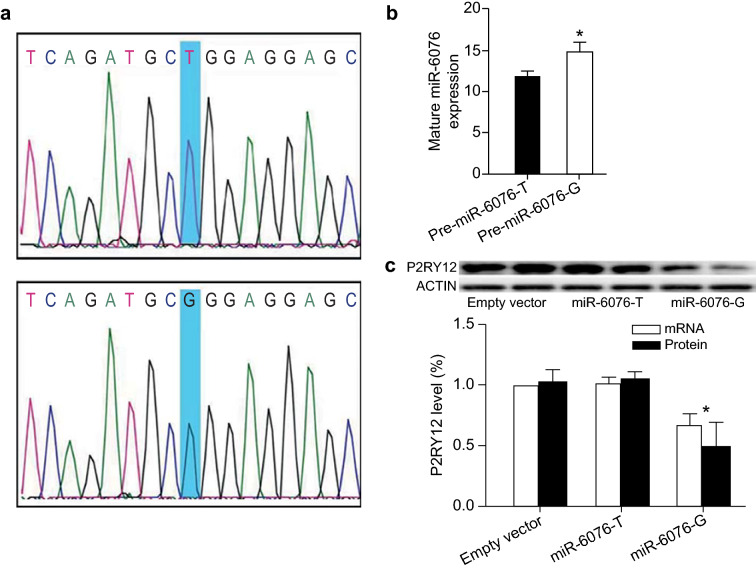
*miR-6076* rs1463411 T/G polymorphisms differentially regulate P2RY12 mRNA and protein levels in cells

**Fig. 4:**
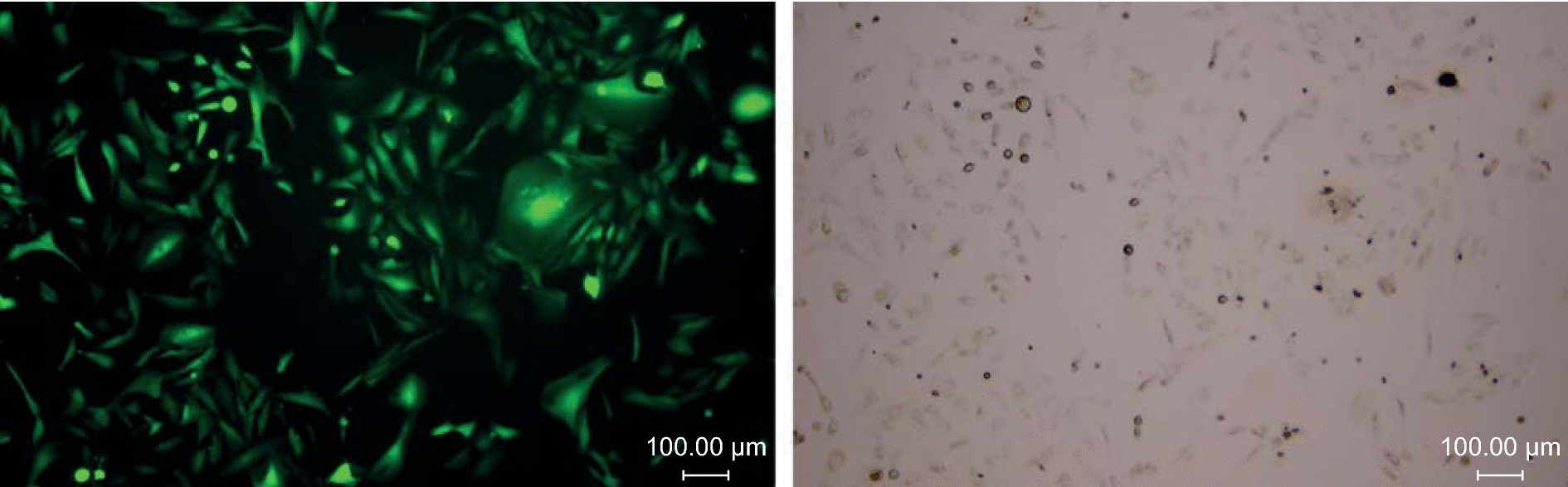
Recombinant lentivirus plasmids containing pre-*miR-6076-T*, pre-*miR-6076-G*, or empty vector used for cell transfection

The recombinant lentiviral plasmids with pre-*miR-6076-T*, pre-*miR-6076-G*, or an empty vector (Fig. 3a) were effectively transfected into BEAS-2B cells, and green fluorescence was observed via fluorescence microscopy (Fig. 4). The processing capability of the pre-*miR-6076-T* plasmid was lower than that of the pre-*miR-6076-G* plasmid (*P* < 0.05; Fig. 3b). The mRNA and protein levels of P2RY12 in cells transfected with pre-*miR-6076-G* were significantly lower than those in cells transfected with *miR-6076-T* (Fig. 3c; *P* < 0.05).

## Discussion

The treatment efficacy and adverse reactions of clopidogrel are two aspects that should be considered by clinicians. However, studies on the effect of miRNAs on the occurrence of bleeding in patients with ACS during clopidogrel treatment are limited. The present study showed that *miR-6076* rs1463411 T/G gene polymorphisms are associated with the risk of bleeding during the first year of clopidogrel treatment in patients with ACS. The allele frequency of rs1463411 G was 16.8%, and it resulted in an increased risk of bleeding. The incidence of bleeding was the highest in patients with the homozygous mutation (GG) and was the lowest in patients with the wild-type TT genotype. This presents a gene-dose effect of *miR-6076* rs1463411 T/G polymorphisms, causing bleeding, the incidence of which increased from 3.1% (TT) to 13.2% (GT) and 27.3% (GG). In addition, a trend of more serious bleeding (BARC 3) occurring in patients with the rs1463411 GT or GG mutation was observed.

Further data showed that *miR-6076* targets the 3ʹ-UTR of *P2RY12* mRNA. In addition, our research demonstrated that the transfection of the pre-*miR-6076-G* plasmid results in increased expression of its mature miRNA compared with that of the pre-*miR-6076-T* plasmid, which is in accordance with the result that the acquisition of the rs1463411 G polymorphism is associated with lower expression of P2RY12 mRNA and protein in cells. Thus, the *miR-6076* rs1463411 G polymorphism could lead to a decreased P2RY12 protein level.

Most protein-coding genes are regulated by miRNAs, and some miRNAs are associated with the progression of cardiovascular diseases [23, 24]. Shi et al. [24] first found that *miR-6076* negatively regulates the expression of the target gene *CHL1*, which participates in the development of some cancers. Based on the biological significance of miRNAs, some miRNA-related polymorphisms have been found to affect drug toxicity. Zhan et al. [25] found that the *miR-196a2* rs11614913 C/T polymorphism alters the expression of specific mature miRNAs and that the toxicity of the drug cisplatin or gemcitabine was higher in CC homozygotes than that in non-CC homozygotes.

The P2RY12 receptor is a critical factor in platelet aggregation. The normal expression of the P2RY12 receptor has been found to be vital in vivo as receptor deficiency in *P2RY12*-KO mice resulted in longer bleeding durations [26, 27]. In this study, the patients with the *miR-6076* rs1463411 G mutation exhibited mild-to-moderate bleeding events during clopidogrel treatment, which may be associated with a lower expression of the P2RY12 receptor in cells upon transfection with plasmids containing* miR-6076-G.*

ACEI or ARB therapy has also been found to be associated with a reduced risk of bleeding during the first year of clopidogrel treatment. ACEI or ARB drugs are important medications for patients with ACS suffering hypertension and subsequent heart failure. A previous study has revealed that ACEI or ARB therapy correlates with a reduced risk of all-cause gastrointestinal bleeding in patients with left ventricular assist devices; this may be due to the inhibition of the angiotensin II signaling pathway and angiogenesis caused by the ACEI or ARB drugs [28]. However, the potential mechanism warrants further study.

The present study also revealed no significant association between CYP2C19*2 or CYP2C19*3 polymorphisms and the risk of bleeding in patients, which is consistent with the findings of a previous study [29]. Bleeding events during clopidogrel treatment are gaining attention. Large-scale research in the future should focus on elucidating the potential mechanisms involving miRNA variants affecting bleeding during clopidogrel therapy in patients with ACS.

The present study has some limitations. First, although we tested 12 gene variants, there are still many other genes and clinical characteristics that may be associated with bleeding events during clopidogrel treatment. Second, 563 Chinese patients with ACS were included in this study; thus, the finding that the *miR-6076* rs1463411 G polymorphism increases the incidence of bleeding events during clopidogrel treatment should be validated in a large population. Third, as this research included only Chinese patients, the results should be confirmed in a Caucasian population.

Bleeding events during clopidogrel treatment are gaining attention in clinical practice. Some patients have to face the contradictory situation of stopping taking antiplatelet drugs when suffering from serious bleeding during clopidogrel treatment, which may increase the risk of cardiovascular events. The present study provides a new perspective to pre-determining the bleeding risk involved during clopidogrel treatment mainly including genetic factors (*miR-6076* variants) and environmental factors (taking ACEI or ARB therapy during treatment), which may benefit patients suffering from ACS. In addition, our study explored the potential mechanism of polymorphism that miR-6076 targets P2RY12 mRNA and that *miR-6076* rs1463411 T/G polymorphisms differentially regulate P2RY12 mRNA and protein levels in cells.

## Supplementary Information

**Additional file 1:** miRNA, CYP2C19*2, and CYP2C19*3 polymorphisms.

## Data Availability

The data used/analyzed in this study cannot be shared publicly owing to the privacy of individuals who participated in the study. The data will be shared on reasonable request to the corresponding author.
