# Supplementary material for: miR-6076 rs1463411 polymorphisms are associated with bleeding during clopidogrel treatment in patients with acute coronary syndrome
Source: Eur J Med Res. 2023 Feb 24;28:96. doi: 10.1186/s40001-023-01068-9 (PMC9951409; doi:10.1186/s40001-023-01068-9)
Supplement: Supplementary file 1 — Additional file 1: miRNA, CYP2C19*2, and CYP2C19*3 polymorphisms. [file 40001_2023_1068_MOESM1_ESM.docx]

***Clinical and Experimental Medicine***

***miR-6076* rs1463411 polymorphisms are associated with bleeding during clopidogrel treatment in patients with acute coronary syndrome**

Zhen-Zhen Mo, Zhen Yuan, Yuan-Yuan Peng, Wan-Lu Zhou, Wei Dai, Guo Wang, Jie Tang, Wei Zhang, Bi-Lian Chen

**Corresponding author**: Bi-Lian Chen

Department of Geriatrics, National Geriatrics Clinic Center, Xiangya Hospital, Central South University

Email: 1281373573@qq.com

**Online Resource 1**. miRNA, *CYP2C19*2,* and *CYP2C19*3* polymorphisms.

| Genetic polymorphism | | MAF (this study) | MAF (HapMap-CHS) |
| --- | --- | --- | --- |
| *CYP2C19*2* | rs4244285 | A 0.291 | A 0.352 |
| *CYP2C19*3* | rs4986893 | A 0.038 | A 0.048 |
| *miR-6076* | rs1463411 | G 0.168 | G 0.219 |
| *miR-4482* | rs45596840 | A 0.134 | A 0.157 |
| *miR-7157* | rs56148568 | C 0.221 | C 0.281 |
| *miR-5186* | rs9842591 | A 0.382 | A 0.538 |
| *miR-7515* | rs10192411 | G 0.074 | G 0.076 |
| *miR-2053* | rs10505168 | C 0.442 | C 0.414 |
| *miR-3612* | rs1683709 | A 0.481 | A 0.481 |
| *miR-499b* | rs2070960 | T 0.179 | T 0.243 |
| *miR-4268* | rs4674470 | C 0.245 | C 0.281 |
| *miR-605* | rs2043556 | G 0.131 | G 0.314 |

MAF: minor allele frequency; CHS: Southern Han Chinese population.
